# Locum doctors in English general practices: evidence from a national survey

**DOI:** 10.3399/BJGP.2023.0039

**Published:** 2023-08-22

**Authors:** Gemma Stringer, Jane Ferguson, Kieran Walshe, Christos Grigoroglou, Thomas Allen, Evangelos Kontopantelis, Darren M Ashcroft

**Affiliations:** Alliance Manchester Business School, Institute for Health Policy and Organisation, University of Manchester, Manchester, UK.; Health Services Management Centre, University of Birmingham, Birmingham, UK.; Alliance Manchester Business School, Institute for Health Policy and Organisation, University of Manchester, Manchester, UK.; Manchester Centre for Health Economics, Division of Population Health, Health Services Research and Primary Care, University of Manchester, Manchester, UK.; Manchester Centre for Health Economics, Division of Population Health, Health Services Research and Primary Care, University of Manchester, Manchester, UK; Danish Centre for Health Economics, University of Southern Denmark, Denmark.; National Institute for Health and Care Research School for Primary Care Research, Centre for Primary Care and Division of Informatics, Imaging and Data Science, University of Manchester, Manchester, UK.; School of Health Sciences, Faculty of Biology, Medicine & Health, University of Manchester, Manchester, UK.

**Keywords:** general practice, locum doctors, national survey, patient safety, quality of care

## Abstract

**Background:**

Locum doctors give practices flexibility to deliver patient services but there are concerns about the impact of locum working on continuity of care, patient safety, team function, and cost.

**Aim:**

To explore locum working in English general practices, and understand why and where locum doctors were needed and how they were engaged, supported, perceived, and managed.

**Design and setting:**

An online survey was sent to 3745 practices.

**Method:**

Quantitative responses were analysed using frequency tables, *t*-tests, and correlations. Free-text responses were analysed using thematic analysis.

**Results:**

In total, 605 (16.2%) responses were returned between June and December 2021. Practices made frequent use of locums, preferring regular locums familiar with processes and patients. Disadvantages of agency locums included cost, lack of patient familiarity, and impact on continuity of care. Care provided by locums was generally viewed as the same but sometimes worse compared with permanent GPs. Some practices reported that locums did not always perform the full range of duties, resulting in increased workload for other staff. Practices were largely unfamiliar with national guidance for organisations engaging locums, and, although processes such as verifying documentation were conducted, far fewer responders reported providing feedback, support for revalidation, or professional development.

**Conclusion:**

Locum working is an essential part of English general practice, but this research raises some concerns about the robustness of arrangements for locum working and the impact on quality and safety of care. Further research is needed about the clinical practice and performance of locums, and to explore how locum working can be organised in ways that assure safe and high-quality care.

## INTRODUCTION

Locum working has benefits for individual doctors and for organisations. For doctors, it may allow greater choice over workload and improved work–life balance,^1^ while for organisations locum doctors provide staffing flexibility. Reports suggest that locum use has increased in recent years and a growing proportion of doctors chose to work as locums,^2^^,^^3^ although recent research suggests that locum use in general practice was fairly stable between 2017 and 2020.^4^

Locums are needed to enable flexibility because of national staff shortages, increases in sickness absence, a high number of doctors leaving the profession early, and, more recently, increased workload and burnout as a result of the COVID-19 pandemic and the workforce crisis.^5^^–^^8^ However, there are concerns about the impact of locum working on continuity of care, patient safety, prescribing, team function, and cost.^9^^–^^11^ The way locum doctors are engaged by organisations may result in higher risk to patient safety; a recent qualitative study found that locums were often perceived to be inferior to permanently employed doctors in terms of quality, competency, and safety, and were frequently stigmatised, marginalised, and excluded.^12^ Research on medical revalidation has highlighted concerns about the rigour of revalidation for doctors working as locums and found rates of deferral of revalidation were higher for locums.^13^ Furthermore, evidence suggests that inadequate support for professional development had implications for clinical governance.^14^

National guidance on how to support locums was produced in 2018 by NHS England.^15^ The guidance emphasises that doctors working as locums can have more difficulty in accessing systems or structures of support. It highlights ways to support locum doctors in providing safe provision of health care. There is some routinely collected data on locum usage in the NHS,^16^ but this tells us relatively little about how often and why locums are needed, and how they are engaged, supported, perceived, and managed.

**Table:** How this fits in

There are concerns about quality and safety when temporary doctors (usually termed locums) are engaged by organisations; however, evidence suggests locums face challenges when they work in unfamiliar environments and organisations need to do more to support locums. The findings from this study indicated that practices were not familiar with NHS England guidance on using locums and support for locums was lacking, particularly with regard to engagement and feedback. The quality of care provided by locums was in some areas reported to be worse than permanent doctors and locums did not cover the same scope of practice as permanent doctors, which can result in more work for other members of the team. Locums are extensively used in general practices and they need to be effectively supported and planned for to enable them to do an effective job, which in turn is likely to improve efficiency and working arrangements for other members of the team.

The authors conducted a national survey of general practices to explore locum work in practices. Information was sought about why and where locum doctors were needed; how locum doctors were engaged, supported, perceived, and managed; and any changes being made in the way locums are used.

## METHOD

### Survey design

The study received research ethics approval from the National Research Ethics Service in England. Agreement to take part was implicit through completion. Stakeholders included in the survey development included: a medical director, a research director, a medical staffing senior manager, chair of the project team’s patient and public involvement and engagement group, a locum GP, and a locum agency managing director. Stakeholder feedback was reviewed by the project team and the survey was adapted accordingly.

An online bespoke questionnaire^17^ consisting of 89 questions was produced using Qualtrics software (version XM). Information was gathered about why locums are required, how they were recruited, supported, perceived, and managed, how locum work compared with permanent doctors, perspectives about use of locum agencies, familiarity with the NHS England and NHS Improvement guidance for supporting locums, and how practices resolved issues. Perspectives were sought regarding the advantages and disadvantages of locums and any future change in locum work (see Supplementary Information S1 for a copy of the survey).

In this study a locum doctor was defined as a doctor in a temporary or fixed-term placement, engaged through a locum agency, GP chambers, or locum bank, or directly contracted by a healthcare organisation.

### Survey distribution

The survey was distributed via the National Institute for Health and Care Research Local Clinical Research Networks (LCRNs). LCRNs’ help increased opportunities for participants to take part in research. This method has been used before and secured a good response rate.^18^ The survey link and reminders were distributed via email to the 14 LCRNs in England who in turn sent it to 3745 practices in their regions (out of 6822 practices in England).

Distribution to practices differed by LCRN, for example, some LCRNs sent the survey link only to practices that had opted to take part in research, whereas others sent it to all practices in their area. In most cases the surveys were sent direct by email to practices but in one region it was distributed via a newsletter.^19^ As a result of the pandemic and to allow practices time to respond, the survey was live from June to December 2021.

### Survey analysis

The survey was analysed using frequency tables to describe numeric and Likert scale data. *t*-tests were performed to compare responder practices with all other practices. Non-parametric tests were used because the majority of survey responses did not show normal distribution. Associations between responses and practice size and frequency of locum use in practices were investigated using Spearman’s correlation coefficient.

Thematic analysis was used to analyse the written responses to three questions on the following areas: the advantages and disadvantages of locum agencies; the advantages and disadvantages of locum work; and the future of locum work.^20^^,^^21^ Patterns of shared meaning were identified using an inductive approach where coding and theme development is based on the information found in the responses.

Responders’ free-text comments were brief and were used to supplement the findings from associated quantitative questions. Familiarisation with the free- text data was achieved through iterative reading and for each question possible codes were noted, taking account of repeated words or meaning.^22^ All written comments were reviewed holistically and overarching themes were mapped, encompassing the key messages across the data. Thematic analysis was not used with one question, asking for perspectives about the NHS England guidance about supporting locums; instead, key descriptive comments were included as examples to provide context to the quantitative question enquiring about practice familiarity with the guidance.

### Responses and characteristics

A total of 3745 practices were surveyed and 605 usable responses were received (response rate 16.2%). The responses included 23 (3.8%) from single-handed practices, 203 (33.6%) from small practices (2–5 GPs), 238 (39.3%) from medium practices (6–10 GPs), and 141 (23.3%) from large practices (>10 GPs). The survey was completed by 205 (33.9%) GP partners, 14 (2.3%) salaried GPs, nine (1.5%) other clinical roles (for example, advanced nurse practitioner), 323 (53.4%) practice managers, 39 (6.4%) non-clinical managers, and 14 (2.3%) administrators; one responder did not complete the job title field.

The response rate (16.2%) was relatively high for an online survey, with research comparing modes of survey administration in a national survey of doctors having found an online response rate of 12.9%.^23^ There was a significant difference in practice list size, GP headcount, locum full-time equivalent (FTE) adjusted for GP FTE (locum use), deprivation, and Care Quality Commission (CQC) ratings between responding practices and all other practices in England. Responding practices were larger in terms of list size and GP headcount but had lower locum use than other practices in England. Responding practices were also more likely to be from a least deprived area and to have better CQC ratings (for further details comparing responding practices and all other practices in England see Supplementary Information S2).

## RESULTS

### The need for locums

#### How often practices use locums

Over half of practices (54.7%; *n* = 331) always (17.0%; *n* = 103) or often (37.7%; *n* = 228) used locums and very few practices made no use of locums (3.1%; *n* = 19). Practices with more GPs employed used locums less frequently than practices with fewer GPs employed, *r*(586) = 0.15, *P*<0.001 (see Supplementary Information S2).

Practices engaged locums for different lengths of time and locums were most frequently engaged on a very short-term basis. Almost one-quarter (23.7%; *n* = 139) of practices needed to engage locums on a long-term basis but one-third (32.6%; *n* = 191) reported never engaging locums long-term (Supplementary Information S2).

#### Reasons for locum use

The main reasons practices reported using locums were to cover planned medical workforce gaps, to provide additional capacity to meet demand or need, and to cover absences due to short-term ill health (Table 1). Practices with fewer GPs employed were significantly more likely to need locums because of difficulties recruiting doctors and less likely to need locums to cover absences as a result of long-term ill health. Practices with higher frequency of locum use were significantly more likely to need locums for all reasons. There were no significant associations between deprivation and reasons for locum use (Supplementary Information S2). There was a statistically significant difference between clinical (GPs and practice nurses) and non-clinical (practice managers and administrators) responders about the reasons for using locums because of difficulties retaining doctors (*z* = −2.12, *P*<0.05) and to cover planned medical workforce gaps (*z* = 2.24, *P*<0.05).

**Table 1.** The reasons practices need to use locums^a^

**Reason**	**Often, *n* (%)**	**Sometimes, *n* (%)**	**Rarely, *n* (%)**	**Never, *n* (%)**	**Associations, correlation coefficient**	
					**Number of GPs employed^b^**	**Frequency of locum use^c^**
Because of difficulties recruiting doctors	154 (26.2)	128 (21.8)	116 (19.8)	188 (32.1)	0.09^d^	0.54^e^
Because of difficulties retaining doctors	45 (7.7)	81 (13.8)	170 (29.0)	290 (49.5)	−0.06	0.33^e^
To cover planned medical workforce gaps	285 (48.6)	222 (37.8)	55 (9.4)	24 (4.1)	−0.04	0.25^e^
To cover absences due to short-term ill health	108 (18.4)	222 (37.8)	165 (28.1)	91 (15.5)	−0.06	0.15^e^
To cover absences due to long-term ill health	62 (10.6)	148 (25.2)	215 (36.6)	161 (27.5)	−0.18^e^	0.19^e^
To provide additional capacity to meet demand or need	169 (28.8)	210 (35.8)	109 (18.6)	98 (16.7)	0.02	0.50^e^

a *Number of responses to the question,* N *= 586.*

b *df = 544.*

c *df = 584.*

d P*<0.05.*

e P<*0.001*.

*df = degrees of freedom.*

#### Factors important to organisations when selecting locums

When selecting a locum to work at their practice, most practices felt that all factors (availability, experience, cost, training, and familiarity with the practice and its patients) were at least moderately important, with slightly greater importance placed on availability and experience, and slightly less importance placed on cost and familiarity. Practices with higher frequency of locum use rated availability as significantly more important *r*(584) = 0.17, *P*<0.001 (Supplementary Information S2).

### How the need for locum doctors is met

The most frequent method to engage locums was using doctors who had previously worked for the practice, followed by word of mouth/personal recommendations. The use of locum chambers and digital platforms such as Locum Nest were much less frequent. The use of locum agencies varied across practices, with just under 40% using agencies often or sometimes but over a third never using them. Practices did not feel that locum agencies consistently matched their needs or provided accurate information. Practices with more GPs employed were both significantly less likely to report that their needs were met by locum agencies *r*(338) = 0.18, *P*<0.001 and that they were provided with accurate information from locum agencies about locums *r*(338) = 0.22, *P*<0.001. Practices with higher frequency of locum use were significantly more likely to have their needs met by locum agencies *r*(367) = 0.19, *P*<0.001 (Supplementary Information S2).

### NHS England and NHS Improvement guidance about supporting locums

Most practices were not familiar with the national guidance about supporting locum doctors, and practice managers and administrators were slightly more familiar with the guidance than GPs and practice nurses (*z* = 2.63, *P*<0.001; Supplementary Information S2). Some felt that it was not applicable to them, not a priority, or they lacked the time to consider it:

*‘I rarely have time to read guidance like this, with so much paperwork being sent to us about more critical issues.’*

(Practice 141)

Some practices followed the guidance or aspects of it and some had their own policies for supporting locums:

*‘I have not read it — we are used to having locums and I have been a PM* [practice manager] *for 13 years, so I feel I/we know what we are doing and support our locums well — we follow our locum policy.’*

(Practice 67)

There were some positive perspectives on the guidance, with some responders reporting it was effective in controlling locum rates and encouraging doctors to take permanent posts:

*‘I think the vision to bring down the rates paid for locum doctors is important as this pushes more into salaried positions and helps provide more stability for both the clinician and practice.’*

(Practice 75)

Whereas others felt that the guidance was unrealistic, impractical, or needed updating:

*‘It is very ivory towers and idealistic, but not practical in real terms.’*

(Practice 7)

#### Application of the guidance

The authors asked practices to tell them how frequently they followed different aspects of the guidance (Table 2). Key procedures such as verifying documentation and induction were conducted more frequently compared with providing feedback or supporting appraisal. Most practices said they would report concerns or complaints about locums.

**Table 2.** Frequency of adherence to the NHS guidance about locums^a^

**When a locum doctor is placed in our organisation we …**	**Always, *n* (%)**	**Often, *n* (%)**	**Sometimes, *n* (%)**	**Rarely, *n* (%)**	**Never, *n* (%)**	**Associations, correlation coefficient**	
						**Number of GPs employed^b^**	**Frequency of locum use^c^**
verify documentation (for example, GMC registration and licence to practise, HPAN, identity, language, health clearance)	516 (94.7)	17 (3.1)	7 (1.3)	2 (0.4)	3 (0.6)	−0.05	0.05
provide an induction to enable them to carry out the work they are being engaged to do, including access to buildings and appropriate IT systems	443 (81.3)	60 (11.0)	33 (6.1)	6 (1.1)	3 (0.6)	−0.01	−0.03
complete an end of placement/exit report	31 (5.7)	49 (9.0)	143 (26.2)	170 (31.2)	152 (27.9)	0.05	−0.02
provide peer/colleague feedback for the locum doctor at the end of the placement	51 (9.4)	84 (15.4)	178 (32.6)	136 (25.0)	96 (17.6)	0.11^d^	0.04
support the locum doctors’ appraisal preparation	49 (9.0)	70 (12.8)	167 (30.6)	136 (25.0)	123 (22.6)	0.03	0.11^d^
provide an annual appraisal for the locum doctor, if appropriate to do so (in light of the nature and duration of the placement)	46 (8.4)	30 (5.5)	100 (18.4)	176 (32.3)	193 (35.4)	0.00	0.09^d^
provide access to professional development activities	84 (15.4)	96 (17.6)	169 (31.0)	106 (19.5)	90 (16.5)	−0.09^d^	0.07
encourage locum doctors to attend multidisciplinary team meetings	126 (23.1)	96 (17.6)	132 (24.2)	106 (19.4)	85 (15.6)	−0.11^d^	0.03
inform the locum doctor and locum agency or RO (where relevant) about serious untoward incidents they have been involved in (even if they are no longer employed at my organisation)	386 (70.8)	48 (8.8)	46 (8.4)	37 (6.8)	28 (5.1)	−0.03	0.03
inform the locum doctor and locum agency or RO (where relevant) about complaints they have been involved in (even if they are no longer employed at my organisation)	375 (68.9)	67 (12.3)	41 (7.5)	30 (5.5)	32 (5.9)	−0.01	0.06
support the locum doctor to engage with revalidation systems within my practice	119 (21.8)	88 (16.1)	146 (26.8)	102 (18.7)	90 (16.5)	0.02	0.06

a *Number of valid responses to the question,* N *= 545.*

b *df = 506.*

c *df = 543.*

d P<*0.05.*

*df = degrees of freedom. GMC = General Medical Council. HPAN = Healthcare Professional Alert Notices. RO = responsible officer.*

### Practice experiences of locum doctors

The practices were asked how the care provided by locums compared with that provided by permanent doctors in a number of areas (Table 3). Generally, care provided by locums was viewed as about the same as or worse than care provided by permanent doctors. Practices employing higher numbers of GPs were significantly more likely to report worse adherence to organisational policies and guidelines, continuity of care, and reporting of adverse events or untoward incidents when care was provided by locums rather than permanent doctors. There was a statistically significant difference between clinical and non-clinical responders about how care was perceived when provided by locums rather than permanent doctors in regard to adherence to organisational policies and guidelines (*z* = 4.56, *P*<0.001), continuity of care (*z* = 6.87, *P*<0.001), avoiding administrative errors (*z* = 4.38, *P*<0.001), reporting adverse events (*z* = 3.12, *P*<0.05), appropriateness of referrals (*z* = 2.87, *P*<0.05), and the functioning of the healthcare team (*z* = 3.27, *P*<0.05; Supplementary Information S2).

**Table 3.** How care is perceived when provided by locums rather than permanent doctors^a^

**Perception**	**Much better, *n* (%)**	**Somewhat better, *n* (%)**	**About the same, *n* (%)**	**Somewhat worse, *n* (%)**	**Much worse, *n* (%)**	**Associations, correlation coefficient**	
						**Number of GPs employed^b^**	**Frequency of locum use^c^**
Adherence to organisational policies and guidelines	8 (1.5)	24 (4.5)	238 (44.8)	236 (44.4)	25 (4.7)	0.15^d^	0.04
Providing continuity of care	7 (1.3)	9 (1.7)	131 (24.7)	274 (51.6)	110 (20.7)	0.21^d^	0.11^d^
Avoiding drug prescribing errors	8 (1.5)	17 (3.2)	405 (76.3)	87 (16.4)	14 (2.6)	0.04	0.08
Avoiding administrative errors	9 (1.7)	13 (2.4)	295 (55.6)	192 (36.2)	22 (4.1)	0.08	0.02
Keeping clear and accurate patient notes/clinical records	13 (2.4)	69 (13.0)	373 (70.2)	69 (13.0)	7 (1.3)	0.07	0.03
Reporting of adverse events or untoward incidents	11 (2.1)	21 (4.0)	374 (70.4)	112 (21.1)	13 (2.4)	0.12^e^	0.06
Appropriateness of referrals	9 (1.7)	8 (1.5)	285 (53.7)	197 (37.1)	32 (6.0)	0.05	−0.05
The functioning of the healthcare team	9 (1.7)	18 (3.4)	325 (61.2)	168 (31.6)	11 (2.1)	0.06	0.05
Workload for permanent members of staff in the healthcare team	20 (3.8)	45 (8.5)	178 (33.5)	212 (39.9)	76 (14.3)	0.06	−0.02

a *Number of responses to the question,* N *= 531*.

b *df = 495*.

c df *= 529*.

d P*<0.001*.

e P<*0.05.*

#### Perspectives about locum doctors and locum agencies

Practices were asked about the advantages and disadvantages of engaging locum doctors and locum agencies, and how they see locum doctor work changing in the future. Maintaining workforce capacity was one of the main advantages reported by responders. Locums filled gaps in the rota and provided cover for sickness, holidays, and maternity leave, which in turn allowed practices to meet patient demand, maintain appointment levels, and relieve workload challenges:

*‘Obvious advantages are to maintain appointment levels and support workload challenges in the event of an absent permanent GP.’*

(Practice 20)

This was particularly important for short- term and short notice cover. Some also felt that locums brought a new perspective and fresh ideas to a practice based on their experiences in different settings. However, some practices noted that locums were not always available when required at short notice:

*‘Locums can bring their own expertise which can be utilised by the practice as they often have “other roles in the NHS”, e.g. minor ops, MSK* [musculoskeletal] *conditions.’*

(Practice 119)

Responders emphasised the advantages of flexibility in the use of locums:

*‘They provide access to large numbers of locums and offer greater flexibility regarding availability and choice of locums.’*

(Practice 102)

They can be used when needed and contracts can be ended easily when they are no longer required. Some practices saw it as more cost-effective to have short-term cover without the longer-term financial commitment of permanent staff:

*‘Can be cost-effective if you have a robust administrative team to support GP admin work. Lack of financial support for paying sick leave, mat* [maternity] *leave etc. and the need to bring in a locum doctor to cover their work when off makes locums a more attractive prospect (as in some cases a salaried doctors’ fees may end up costing a practice up to 30% more than the base salary you’d pay a locum).’*

(Practice 75)

Some thought locum agencies were efficient in sourcing locums and doing all necessary pre-employment checks and paperwork required.

On the other hand, lack of familiarity with the practice, the area, the patients, and local referral pathways was a disadvantage for practices. It was time consuming for practices to get a new locum set up on all the practice systems. Not knowing the locum resulted in uncertainty about the quality of their work, their efficiency, and whether they would create more workload:

*‘You end up with another new locum who doesn’t know the practice or the patients. New locums take time as there is paperwork and IT set-up to do, checking CVs and certificates etc. You don’t know what you’re getting, how quickly they work, whether they’re good or whether they create more work for the GPs to come back to.’*

(Practice 171)

GP practices preferred to work with locums that were familiar with the practice and its patients as this meant that the locum was able to get on with the job and also meant the benefit of continuity of care for patients:

*‘If you are able to have the same GPs come back to you for the next periods … they become more familiar with surgery policies etc. and are increasingly autonomous in their work.’*

(Practice 99)

*‘Having regular locums helps with patient services and continuity of care for patients.’*

(Practice 218)

However, continuity was likely to be shorter term and episodic. Consequently, locum use was generally considered to have a negative impact on continuity of care and this was particularly problematic for patients with long-term or complex conditions:

*‘Can be more difficult when trying to achieve continuity and they often work at a slower pace.’*

(Practice 27)

*‘Good to see patients for acute on-the- day conditions, not so great for long-term problems and conditions.’*

(Practice 138)

Cost was one of the main disadvantages reported about locum agencies. Responders felt that locum agencies control the market and drive up rates:

*‘Signing up most regional locums to mainly one agency reduces competition between individual locums and results in high locum rates, and high locum rates in turn affect the affordability of locums and reduce the likelihood of locums ever again joining the workforce as salaried GPs or GP partners.’*

(Practice 102)

For some, the use of locum agencies to source a locum was a last resort because of the additional costs. The view that locums are expensive contributed to a negative view of locums and created tensions between locums and permanent staff:

*‘Many* [locum] *GPs are charging astronomical fees which are not appropriate for the work they are doing, yet often surgeries have no choice. I think this can create ill feeling.’*

(Practice 104)

High locum pay rates were also considered to be a reason why locums would not join the permanent workforce:

*‘I think more and more GPs will turn to locum work rather than regular work as the money is better and the terms of work are better as they can take time off when they want and do the hours they prefer.’*

(Practice 2)

*‘I hope that locums would be regulated with regard to charges, as demand often means that practices do not have any choice with regard to how much they have to pay locums. Locums can, therefore, earn much more than GP partners or salaried GPs and specify clinic sizes and times worked, which does not encourage them to take permanent posts in practices.’*

(Practice 119)

Responders felt that the use of locums would continue to increase because of the higher pay, lower workload, and greater autonomy. To encourage locums to take up permanent posts some responders wanted stricter regulation of locum pay and access to pensions. Another suggestion was the use of a pool of locums employed locally to reduce costs and improve familiarity and continuity of care.

Responders reported that locums generated increased workload for other practice staff, particularly administrative work. The way that locums negotiate terms and conditions was felt to result in them not always performing the full range of duties, for example, not doing administrative work. Other reasons for increased workload included high referral rates, differences in prescribing practices, and locums asking patients to return for another appointment. Sometimes patients would refuse to see a locum or would return to see their regular GP after seeing a locum because they were dissatisfied:

*‘Some locums just defer work telling patients to call back another day or prescribe in a way we don’t.’*

(Practice 168)

*‘Disadvantages: non-clinical workload, issues passed down the line for later and not sorted out, increased clinical and non-clinical burden on permanent team, expense, adherence to protocols, medicines optimisation, referrals, lack of familiarity with local services can increase workload for GPs, limits to workload (e.g. no visits, no duty Dr, no cover, won’t work alone), continuity, permanent Drs “picking up the pieces”.’*

(Practice 61)

For some, locums were viewed positively but for others locums were seen as a last resort and did not present a long-term solution to staffing problems:

*‘Some locums are very good and work hard.’*

(Practice 2)

*‘But in most other respects e.g. continuity of care, working within the MDT* [multidisciplinary team]*, being aware of local policies etc., being able to help with admin in the practice, they are not as good.’*

(Practice 136)

*‘They are a sticking plaster only really.’*

(Practice 55)

There was a perception that locums were not invested in the practice or team. Examples of this included not contributing to quality improvements and achieving targets:

*‘They have no loyalty to the surgery or patients.’*

(Practice 67)

In order for locums to have a better understanding of general practice some wanted locums to have a permanent position either before or during their locum work, and felt that this would provide locums with a community of practice and improve team working and shared workload:

*‘I consider different regulation is needed, and doctors who want to do locums should have also a regular job — perhaps one day a week based in one place — to understand better general practice, continuity of care, team work, sharing workload, importance of coding, of electronic health records and problem lists maintenance.’*

(Practice 77)

### Dealing with concerns about locums

Practices were asked to state what happened when there were low-, medium-, or high-level concerns about a locum doctor in their practice. Low-level concerns were defined as causing no harm to patients or staff and the doctor was not at any personal risk; medium-level concerns have potential for serious harm to patients or staff, or the doctor was at personal risk; and high-level concerns are when patients, staff, or the doctor had been harmed. The action that practices reported they would take to deal with concerns increased depending on the severity of the concern (Table 4). The higher the severity of the concern the more likely that locums and locum agencies would be informed. Practices reported that it was common for contracts to be ended early when there were concerns and locums not to be used again.

**Table 4.** How practices deal with different levels of concern about locums^a^

**Level of concern**	**Always, *n* (%)**	**Most of the time, *n* (%)**	**About half the time, *n* (%)**	**Sometimes, *n* (%)**	**Never, *n* (%)**	**Associations (correlation coefficient)**	

						**Number of GPs employed^b^**	**Frequency of locum use^c^**
**Low-level concern**							
The locum doctor is informed	262 (51.8)	131 (25.9)	32 (6.3)	73 (14.4)	8 (1.6)	0.06	0.05
Reported to the locum agency	153 (30.2)	91 (18.0)	26 (5.1)	125 (24.7)	111 (21.9)	0.13^d^	0.08
Reported to the GMC	35 (6.9)	20 (4.0)	20 (4.0)	136 (26.9)	295 (58.3)	0.11^d^	−0.05
The locum contract is ended early	58 (11.5)	49 (9.7)	30 (5.9)	231 (45.7)	138 (27.3)	0.05	0.02
We would not use that locum again	136 (26.9)	115 (22.7)	47 (9.3)	166 (32.8)	42 (8.3)	−0.04	−0.03

**Medium-level concern**							
The locum doctor is informed	414 (81.8)	63 (12.5)	12 (2.4)	12 (2.4)	5 (1.0)	0.02	0.02
Reported to the locum agency	308 (60.9)	81 (16.0)	23 (4.5)	35 (6.9)	59 (11.7)	0.06	0.09
Reported to the GMC	111 (21.9)	83 (16.4)	47 (9.3)	158 (31.2)	107 (21.2)	−0.01	−0.00
The locum contract is ended early	188 (37.2)	120 (23.7)	51 (10.1)	120 (23.7)	27 (5.3)	−0.03	0.01
We would not use that locum again	280 (55.3)	101 (20.0)	35 (6.9)	76 (15.0)	14 (2.8)	−0.06	−0.03

**High-level concern**							
The locum doctor is informed	485 (95.8)	9 (1.8)	5 (1.0)	1 (0.2)	6 (1.2)	−0.04	0.01
Reported to the locum agency	433 (85.6)	16 (3.2)	8 (1.6)	4 (0.8)	45 (8.9)	0.03	0.03
Reported to the GMC	377 (74.5)	45 (8.9)	20 (4.0)	41 (8.1)	23 (4.5)	−0.01	−0.03
The locum contract is ended early	398 (78.7)	45 (8.9)	16 (3.2)	30 (5.9)	17 (3.4)	−0.04	−0.02
We would not use that locum again	422 (83.4)	35 (6.9)	11 (2.2)	22 (4.3)	16 (3.2)	−0.04	−0.00

a *Number of responses to the question,* N *= 506*.

b *df = 472.*

c *df = 504.*

d P*<0.05.*

*df = degrees of freedom. GMC = General Medical Council.*

## DISCUSSION

### Summary

This study found that the use of locums was a common and essential part of practice working. The use of locums was motivated by workforce issues such as recruitment, providing capacity to meet demand, and planned workforce gaps. However, it was found that there was poor awareness of and some confusion about the national guidance for locums and who was responsible for following it. Lack of familiarity with national guidance could result in locums receiving less support and integration into the practice and could be a barrier for locums to work effectively. Practices focused on key processes, such as registration checks and induction, but much less was done with regard to engagement, feedback, and appraisal.

### Strengths and limitations

There were some differences between responding practices and all other practices in England, and so the results should be interpreted with caution. This could be a consequence of sampling research active practices via the LCRN.

The views and perceptions collected in this survey will vary depending on both the representativeness of the responders and their awareness of the topics covered. GP partners and practice managers would be expected to have a good understanding of locum work and management, but responders in other roles may not have sufficient experience and knowledge to answer all questions accurately. Responses are also the views of just one person in the practice and may differ from the views of others in the same practice.

### Comparison with existing literature

The pressures of the current workforce and workload crisis in general practice^24^ may affect practices’ ability to provide the time and resources to support locums. Failing to provide support with appraisal and feedback for locums could result in locums feeling marginalised and excluded,^12^ which in turn does not contribute towards goals of improved patient safety and collaboration.^25^

This study found that practices generally thought that the quality of care provided by locums was the same as or worse than that provided by permanent GPs, particularly in areas such as continuity of care, adherence to policies and guidelines, and making appropriate referrals. Common operational problems that afflict GPs (such as difficulties with computer systems, problems with equipment, and challenges with the coordination of care)^26^ may affect locums more because of their lack of familiarity with practice systems and processes. Although practices felt that continuity of care was worse when care was provided by a locum, it should be recognised that in some practices traditional ideas of relational continuity have already been eroded and replaced in part by managerial and informational continuity.^9^^,^^27^^–^^29^ Practices preferred not to use locum agencies and would rather recruit known and familiar locums. There were low opinions of locum agencies, particularly in relation to cost and a general perception that locums were expensive. Although this study found that practices wanted to see greater control of locum pay caps and reduced incentives for locum work, it is important to note that broader discontent with GP pay and conditions^30^ may have contributed to the current recruitment and retention challenges.^31^

Contrary to job demands–resources theory,^32^ practices reported that the use of locums increased workload for permanent staff, particularly in non-clinical and administrative roles. Certain tasks may not be done by locums because they are unfamiliar with the work setting, it is expensive to pay them to do administrative work, or they are not there to follow up, so other staff are required to pick up the work.

### Implications for research and practice

Locum work is precarious and contracts can be terminated early following even low- level concerns. This is a way for practices to maintain safety and patient satisfaction, but as a consequence the locum is not supported and performance issues may not be remedied before the locum moves to the next practice.^33^ This may in part explain why locums are more likely to have formal complaints made about them to the professional regulator than permanent doctors.^2^ Doctors who are employed may be required by the General Medical Council to undergo remediation if there are concerns about their practice.^34^ However, locum doctors are not in a conventional employed relationship and may find it difficult to access support for remediation if it is indicated.

The CQC may review locum induction arrangements and credential checking during inspections.^35^ Practices should consider how they can enable locums to follow local prescribing guidelines, such as those for antibiotic prescribing regimes.^36^

In conclusion, although these findings show that locum working is a common and necessary part of English general practice, it raises a number of concerns about how locums are integrated and supported, and how this has an impact on the quality and safety of care. Practices could consider how they might better integrate locums into organisational systems, give guidance on processes, and communicate effectively, particularly regarding any concerns. Further research is needed to compare empirically the clinical practices and performance of locum and permanent GPs, and to suggest how practices (and others such as primary care networks and the newly created integrated care systems) can organise locum working in ways that assure the quality and safety of care.

## Data

The data that support the findings of this study are available from the corresponding author, on reasonable request.
